# Hacking Cough

**Published:** 1864-10

**Authors:** 


### HALL'S JOURNAL OF HEALTH.

VOL. XI. OCTOBER, 1864. No. 10.

### HACKING COUGH.

It is not known as generally as it ought to be, that consumption, that disease known as "common consumption of the lungs," called by many a "decline," begins with a regular hacking cough in the morning, or on getting up. In fact it is so slight at first, that it scarcely amounts to a cough; it is a mere "hem" or effort to clear the throat of something which seems to excite a little tickling sensation there, generally referred to the spot known as the hollow, at the bottom of the neck in front. The person does not imagine that it has any thing whatever to do with the lungs; he insists upon it that it is only in the throat; and for weeks and even months afterwards, when it has become a decided cough, as regular as the rising of the sun, he regards it as an affection of the throat merely, and repeats the saying a thousand times, as if to reassure himself and others of the truth of it; and that if he could only get something to apply to the throat, and take away "that pesky tickling," he would have no cough, and would be as well as he ever was in his life; he strikes upon his chest triumphantly, and exclaims "all right, not the slightest pain or other inconvenience there, anyhow." To remove this tickling he has the fashionable remedy applied; a bit of sponge is dipped into a solution of the nitrate of silver, and passed down to the tickling spot, and lo it is gone! To make the matter more sure, the applications are renewed daily or several times in the course of a week or two; the physician is gratefully and liberally paid and he returns home with a heart so light and buoyant, he is ready to hug and kiss everybody he meets; is quite as jubilant as an honest man, who by great and long continued exertions, has paid the last dollar of indebtedness he owes in the world. In about two years, on an average, the throat swabbed man dies of consumption; because the nitrate of silver only destroys the sensibilities of the part by the greater wounding inflicted, when nature has recovered from the violence offered, the tickling returns, just the same as ever, and then there is the usual routine of syrups, cough drops, lozenges, trochees and the like, all of which contain opium, which like the nitrate of silver, only deadens the sensibilities of the parts, but for a short period, requiring renewals every few hours; then follow the more desperate (and as vain) efforts of going to the South, to a warmer climate, or Minnesota, to die in some miserable log cabin, away off in some cheerless prairie, or bleaker " thicket," or in some more pretentious " water cure." Whole Hecatombs of the dead are piled up every year in this way from one mistaken idea. The cause of the tickling in the throat is tubercles in the lungs; the throat is the point where we get the intelligence of their beginning existence. A tubercle is of the size of the tiniest crust of bread, which, when it " goes the wrong way," that is passes into the lungs, is a foreign matter there, and so is the tubercle; nature takes the alarm, and endeavors to get rid of it, by exciting a tickling in the throat, which brings about a cough, and this cough generally, as all have experienced, continues until the crumb is dislodged. Destroying the tickling feeling here, is to remove the kindly cough, intended to bring the offending crumb away. The time to " cure consumption," is when it has proceeded no farther than the morning cough; the manner of doing it is to remove, to cause absorption of, or otherwise rendering the tubercle harmless, by developing the activities of the lungs, by correcting that "error of nutrition" which the most eminent physiologists of the age agree in regarding as the cause of tubercle; and let the tickling and the throat and the cough alone, as the friendly sentinels of danger: when the tubercle is rendered harmless, the throat will be well, and the cough and tickling will disappear. Defective nutrition is to be corrected by keeping the system in the highest state of health possible; in the breathing of large amounts of out door air; in the consumption of nutritious food; and the working out of all impure matters from the system by moderate, continuous, interesting and profitable activities; all the while aiding nature, by such means as the scientific practitioner well understands, will keep the skin, liver, feet, and the whole digestive apparatus in the most perfect order, without which, the rendering of tubercles harmless, that is, the arrest of comsumption, is absolutely and always, utterly impossible.

